# Supplementary material for: Prospects for a sequence-based taxonomy of influenza A virus subtypes
Source: Virus Evol. 2024 Aug 17;10(1):veae064. doi: 10.1093/ve/veae064 (PMC11378807; doi:10.1093/ve/veae064)
Supplement: veae064_Supp [file veae064_supp.zip › suppl_data/suppmat.pdf]

Prospects for a sequence-based taxonomy of influenza A virus subtypes: Supplementary Materials

Art F. Y. Poon[1,2,3]

[1]Department of Pathology & Laboratory Medicine, Western University, London, Canada

[2]Department of Microbiology & Immunology, Western University, London, Canada

[3]Department of Computer Science, Western University, London, Canada

Supplementary Figures

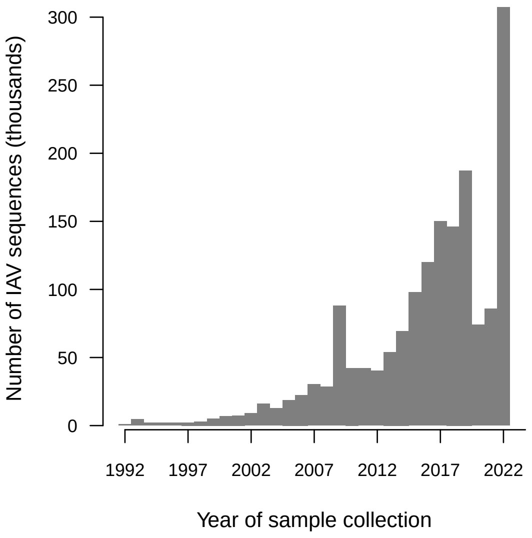

Figure S1: Summary of the number of influenza A virus sequences in the GISAID database by year of sample collection. Note that some sequences were collected prior to the establishment of this database in 2006, and submitted retrospectively at a later date. The number of sequences per isolate varies not only because of varying numbers of segments sequenced, but also because some isolates were sequenced more than once. This trend features a conspicuous surge in the number of sequences associated with the 2009 H1N1 pandemic, as well a drop in numbers in 2019-2020 in association with the SARS-CoV-2 pandemic. Overall, the number of sequences is exponentially growing over time (adjusted $R^2 = 0.926$).

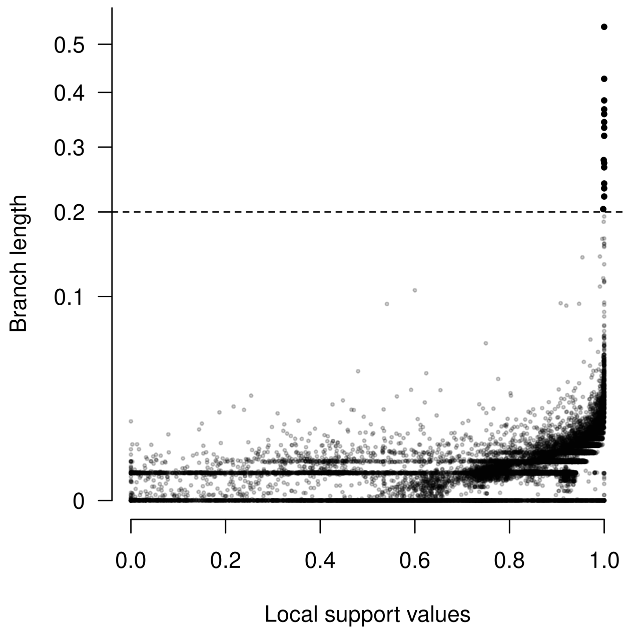

Figure S2: A scatterplot comparing branch lengths and local support values for internal nodes of a maximum likelihood tree relating HA sequences from this study. Local support values are calculated by the Fasttree program using the Shimodaira-Hasegawa test Price et al. 2010. These values and the associated branch lengths are significantly correlated (Spearman's $\rho = 0.807$, $P < 10^{-12}$). The longest branches (*e.g.*, 16 branches with lengths above 0.2, dashed line) that are most likely to separate the tree into different subtypes are almost entirely associated with support values of 1.0.

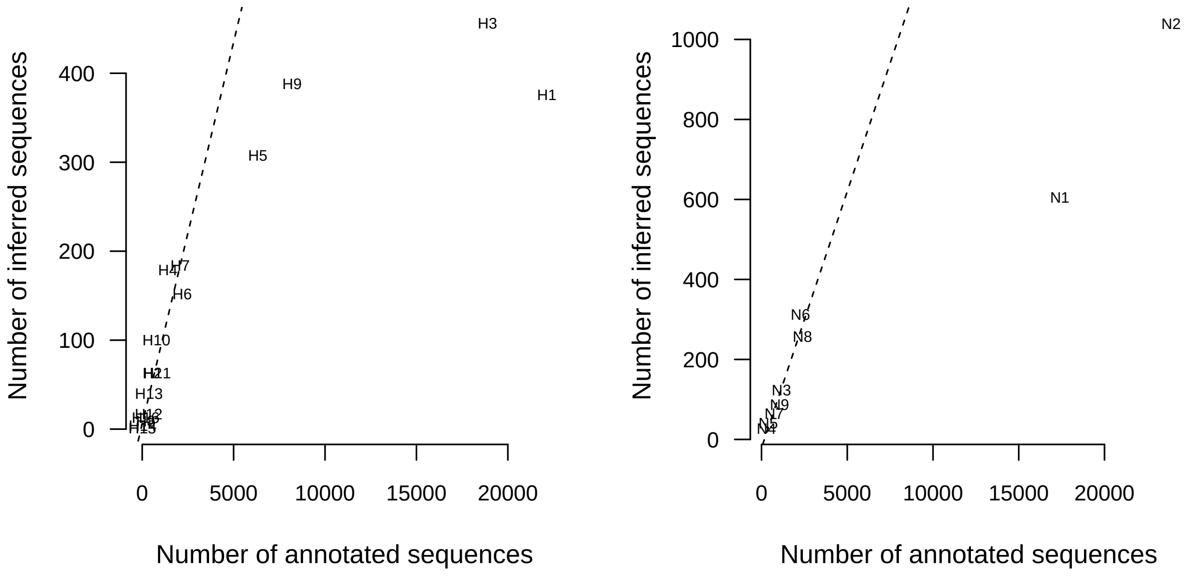

Figure S3: Influenza A virus hemagglutinin (HA, left) and neuraminidase (NA, right) sequences corresponding to the most common subtypes are disproportionately less likely to be unannotated. Each scatterplot displays the numbers of inferred subtypes (y-axis) for unannotated HA or NA sequences (*i.e.*, labelled 'unknown', 'HX' or 'mixed') compared to their respective numbers in annotated sequences (x-axis). Subtypes were inferred by retrieving each sequence's nearest neighbour in the phylogeny. Dashed lines represent linear regressions on counts excluding the most common subtypes (H1, H3, H5 and H9 for hemagglutinin; N1 and N2 for neuraminidase).

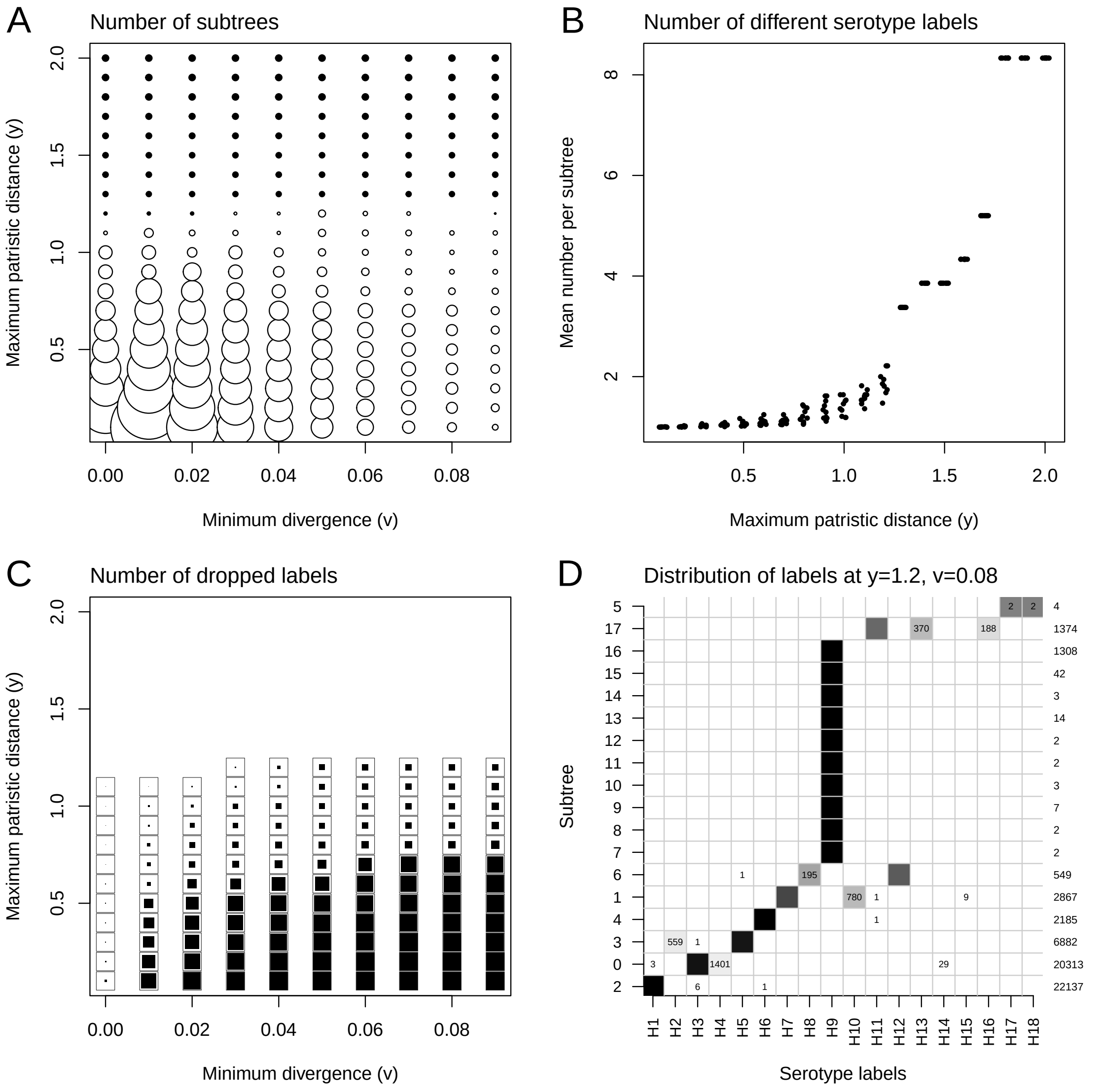

Figure S4: Evaluation of concordance between HA subtype annotations (labels) and subtrees under varying thresholds of nodewise clustering, for comparison to edgewise clustering results summarized in Figure 2. (A) The size of each point is scaled to the discordance between the number of subtrees and the target number ($n = 18$), *i.e.*, smaller is better. Open circles indicate too many subtrees, and filled circles indicate too few. (B) The number of different labels increases as a function of the mean patristic distance cutoff (y). The ideal number is one label per subtree. Varying the cutoff on minimum divergence (v) had a limited effect on this outcome. (C) The proportion of labels that are not associated with any subtree at given cutoffs are represented by filled squares. For reference, an outline is drawn for each square to represent 100% loss. (D) The distribution of labels among subtrees defined by cutoffs at which the ideal number of subtrees is obtained ($y = 1.2$ and $v = 0.08$). Regions are shaded in proportion to the fraction of each label in the subtree, and the number of labels is displayed if the fraction is ≤ 0.5. The total number of labels per subtree is displayed along the right margin.

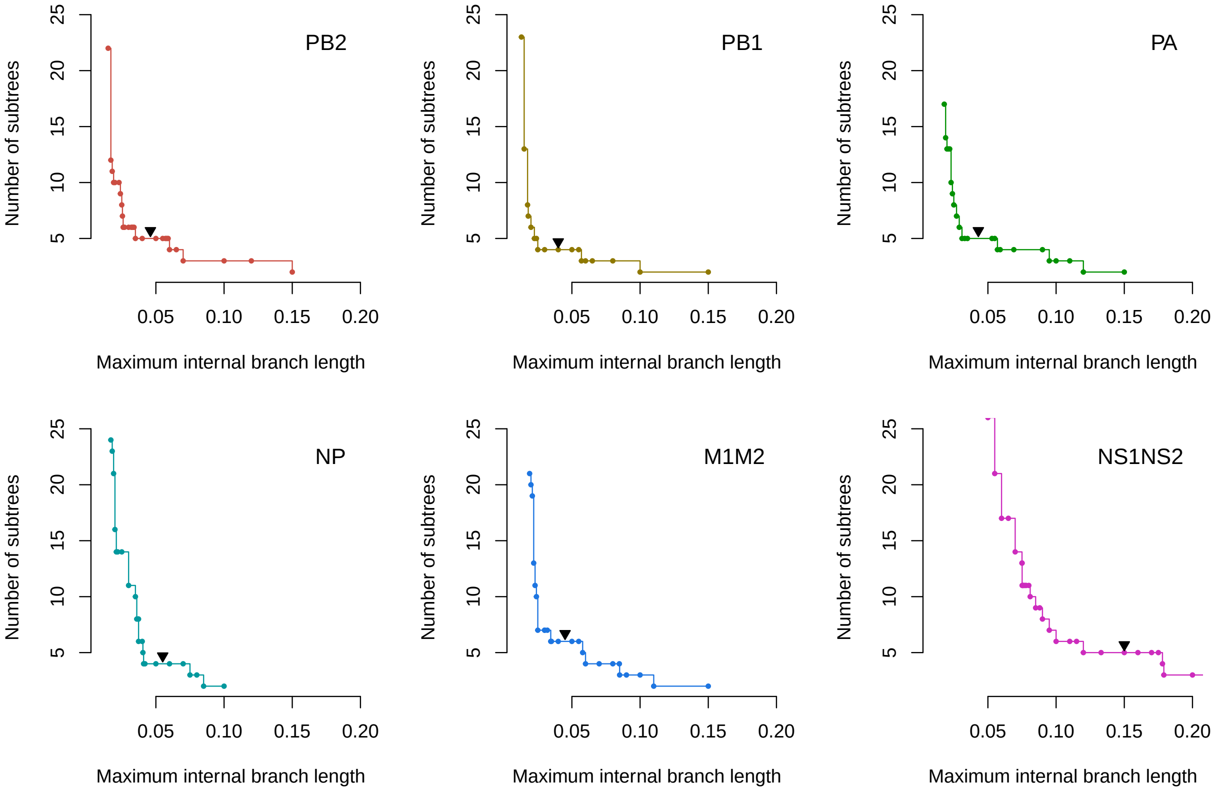

Figure S5: Number of subtrees as a function of internal branch length cutoffs applied to the phylogenies of other proteins. The plots are ordered with respect to genome segment numbering, *e.g.*, protein PB2 is encoded by segment 1. Solid triangles mark the cutoffs where the composition of the resulting subtrees are summarized in Supplementary Table S4. Since the edgewise clustering method yields substantially fewer subtrees for these phylogenies than HA and NA, we cannot use the same approach of taking the longest segment for the most robust number of subtrees. Instead, I manually selected the first long segment past the subjective 'elbow' for each trend. As shown in Supplementary Table S4, changing this cutoff has little impact on the composition of subtrees.

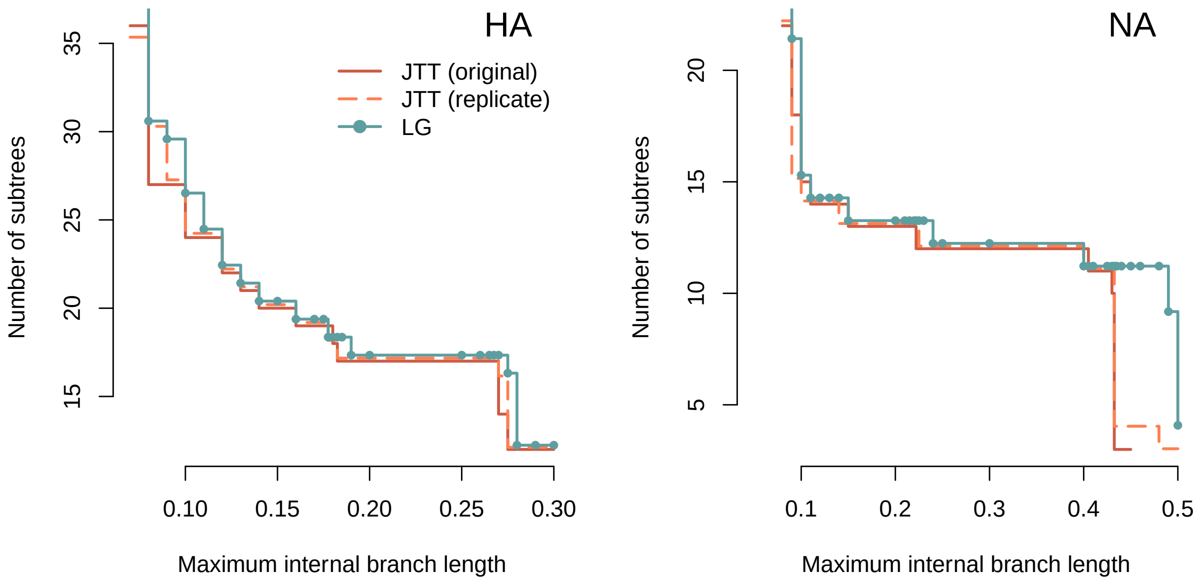

Figure S6: Modified versions of Figures 2A and 3A with the addition of lines and points (solid, blue) indicating the number of subtrees produced by partitioning at varying branch length thresholds of the phylogenies reconstructed from HA or NA sequences under the Le-Gascuel model of amino acid substitutions. The original trends derived from trees reconstructed under the default Jones-Taylor-Thornton (JTT) model are reproduced in orange (solid). In addition, a second trend (light orange, dashed) derived from replicate JTT trees is drawn to illustrate the stochastic variation in FastTree outputs.

Supplementary Tables

Segment	Gene products	Min. length	Total	Unique
1	PB2	2000	93,415	32,718
2	PB1, *PB1-F2*	2000	160,506	29,790
3	PA, *PA-X*	2000	169,484	32,349
4	HA	1600	137,172	66,874
5	NP	1400	103,033	18,978
6	NA	1000	126,233	52,146
7	M1, M2	900	213,717	14,389
8	NS1, NS2	750	191,676	25,172

Table S1: Summary of amino acid sequences obtained for all eight IAV segments from the NCBI Genbank nucleotide database (date accessed, June 1, 2023 except for HA sequences, which were retrieved April 28, 2023). Amino acid sequences representing PB1-F2 and PA-X (italics) were excluded from subsequent analyses. The M1/M2 and NS1/NS2 sequences were concatenated with amino acid residues within overlapping regions excluded. Min. length = minimum sequence length in nucleotides. Total = number of sequence records returned by query. Unique = number of unique amino acid sequences after filtering.

Accession	Strain	Original	Corrected
ON427060	A/swine/Kansas/A02711700/2022	H1	H3
MF098827	A/swine/Chile/VN1401-587/2014	H1	H3
MT377993	A/swine/Thailand/CU22337/2018	H1	H3
MH596892	A/ruddy turnstone/Delaware/650622/2002	H11	H7
MN569599	A/Newcastle/177/2017	H3	H1
MN574095	A/Victoria/79/2017	H3	H1
KM110063	A/swine/Hanoi/568/2013	H3	H1
KM110062	A/swine/Hanoi/424/2013	H3	H1
KM110061	A/swine/Hanoi/411/2013	H3	H1
CY251407	A/Victoria/1003A/2012	H3	H1
OP888980	A/yellow-billed	H6	H1
LC500374	A/duck/Vietnam/HU8-1918/2017	H3	H5
MH598081	A/ruddy turnstone/New Jersey/650677/2002	H11	H6
MH598105	A/ruddy turnstone/New Jersey/828212/2001	H5	H12
MH130194	A/mallard/Korea/M198/2013	H4	H9

Table S2: Reclassification of IAV HA sequences with serotype (subtype) annotations that are not consistent with their location in the phylogeny. Discordant sequences were detected as outliers in box-and-whisker plots of their vertical positions in the rectangular layout of the tree, grouped by subtype annotations. The predicted subtypes were estimated from the nearest labeled tips in the tree. These predictions were manually verified using nucleotide BLAST.

Accession	Strain	Original	Corrected
MK960206	A/Texas/9437/2019	N1	N2
OP940105	A/swine/Iowa/G92995/2022	N1	N2
OP645900	A/swine/Iowa/C61728/2022	N1	N2
OP940090	A/swine/Illinois/E80174/2022	N1	N2
MW839927	A/swine/Iowa/CD81015/2015	N1	N2
MW839656	A/swine/North Carolina/LJ8316/2016	N1	N2
MT377995	A/swine/Thailand/CU22337/2018	N1	N2
MF099208	A/swine/Chile/VN1401-587/2014	N1	N2
MT378776	A/swine/France/22-180459/2018	N1	N2
MW362711	A/swine/Belgium/Gent-36/2016	N1	N2
MT818840	A/Mallard/North Carolina/AH0114261I.6.A/2017	N8	N2
MW513533	A/green-winged teal/Alaska/UGAI16-4602/2016	N8	N2
MF613906	A/ruddy turnstone/Delaware Bay/303/2016	N4	N2
MH597119	A/ruddy turnstone/Delaware/1016391/2003	N5	N2
MH597724	A/ruddy turnstone/New Jersey/1321395/2005	N8	N6
MH597785	A/ruddy turnstone/New Jersey/1321408B/2005	N8	N6
MH598073	A/ruddy turnstone/New Jersey/650675/2002	N9	N5
MH597447	A/ruddy turnstone/Delaware/650647/2002	N9	N5
L06581	A/equine/New Market/1979	N3	N8
MH597692	A/ruddy turnstone/New Jersey/1148671/2004	N7	N8
MH596859	A/ruddy turnstone/Delaware/1016396/2003	N9	N8
LC431470	A/pintail/Alberta/121/1979	N8	N4
CY251409	A/Victoria/1003A/2012	N2	N1
MH598004	A/ruddy turnstone/New Jersey/650584/2002	N9	N1
MH596894	A/ruddy turnstone/Delaware/650622/2002	N4	N1
MH579375	A/wild waterfowl/Korea/M54-F-1/2010	N2	N1
MK636253	A/New York/7745/2018	N2	N1
ON848942	A/Homosapiens/India/149/2017	N2	N1
MN568417	A/Brisbane/144/2017	N2	N1
MN574097	A/Victoria/79/2017	N2	N1

Table S3: Reclassification of IAV NA sequences with serotype (subtype) annotations using the same methods described in Table S2.

Protein	# tips	Example strain	HnNn
PB2	32,700		
	4	A/equine/Prague/1/1956	H7N7
	4	A/dark fruit-eating bat/Bolivia/PBV780-781/2011	H18N11
	2	A/Bat/Egypt/381OP/2017	H9N2
	2	A/little yellow-shouldered bat/Guatemala/164/2009	H17N10
PB1	29,685		
	5	A/equine/Prague/1/1956	H7N7
	4	A/flat-faced bat/Peru/033/2010	H18N11
	2	A/little yellow-shouldered bat/Guatemala/164/2009	H17N10
PA	32,325		
	3	A/equine/Prague/1/1956	H7N7
	3	A/dark fruit-eating bat/Bolivia/PBV780-781/2011	H18N11
	2	A/Bat/Egypt/381OP/2017	H9N2
	2	A/little yellow-shouldered bat/Guatemala/164/2009	H17N10
NP	18,970		
	4	A/dark fruit-eating bat/Bolivia/PBV780-781/2011	H18N11
	2	A/Bat/Egypt/381OP/2017	H9N2
	2	A/little yellow-shouldered bat/Guatemala/164/2009	H17N10
M1/M2	14,367		
	12	A/equine/Lexington/1/1966	H7N7
	3	A/swine/Western Australia/AS12-2111-02/2012	H3N2
	3	A/Artibeus lituratus/Brazil/2344/2012	H18N11
	2	A/Bat/Egypt/381OP/2017	H9N2
	2	A/little yellow-shouldered bat/Guatemala/164/2009	H17N10
NS1/NS2	23,380		
	4	A/equine/Prague/1956	H7N7
	2	A/Bat/Egypt/381OP/2017	H9N2
	2	A/little yellow-shouldered bat/Guatemala/164/2009	H17N10
	2	A/flat-faced bat/Peru/033/2010	H18N11

Table S4: Composition of subtrees produced at selected branch length cutoffs for phylogenies relating other protein sequences. Cutoffs were chosen based on the trends displayed in Supplementary Figure S5. Example strain and HA/NA subtype labels (HnNn) are omitted for the largest subtrees.
